# GPCRdb in 2018: adding GPCR structure models and ligands

**DOI:** 10.1093/nar/gkx1109

**Published:** 2017-11-16

**Authors:** Gáspár Pándy-Szekeres, Christian Munk, Tsonko M Tsonkov, Stefan Mordalski, Kasper Harpsøe, Alexander S Hauser, Andrzej J Bojarski, David E Gloriam

**Affiliations:** Department of Drug Design and Pharmacology, University of Copenhagen, Universitetsparken 2, DK-2100, Copenhagen, Denmark; Department of Medicinal Chemistry, Institute of Pharmacology, Polish Academy of Sciences, Smetna 12, 31-343 Krakow, Poland

## Abstract

G protein-coupled receptors are the most abundant mediators of both human signalling processes and therapeutic effects. Herein, we report GPCRome-wide homology models of unprecedented quality, and roughly 150 000 GPCR ligands with data on biological activities and commercial availability. Based on the strategy of *‘Less model – more Xtal’*, each model exploits both a main template and alternative local templates. This achieved higher similarity to new structures than any of the existing resources, and refined crystal structures with missing or distorted regions. Models are provided for inactive, intermediate and active states—except for classes C and F that so far only have inactive templates. The ligand database has separate browsers for: (i) target selection by receptor, family or class, (ii) ligand filtering based on cross-experiment activities (min, max and mean) or chemical properties, (iii) ligand source data and (iv) commercial availability. SMILES structures and activity spreadsheets can be downloaded for further processing. Furthermore, three recent landmark publications on GPCR drugs, G protein selectivity and genetic variants have been accompanied with resources that now let readers view and analyse the findings themselves in GPCRdb. Altogether, this update will enable scientific investigation for the wider GPCR community. GPCRdb is available at http://www.gpcrdb.org.

## INTRODUCTION

G protein-coupled receptors represent both the largest membrane and drug target protein families, accounting for 800 (4%) human genes and 34% of FDA-approved drug targets (1). The human GPCRs have been divided into six classes (or families): A (Rhodopsin), B1 (Secretin), B2 (Adhesion), C (Glutamate), F (Frizzled) and Taste 2 based on evolutionary homology (2,3). All GPCRs share a common heptahelical transmembrane domain carrying the signal from the extracellular ligand to the intracellular signalling protein, typically one of four families of G proteins or β-arrestin, which can also internalise the receptor. Recently, structural biology and pharmacology breakthroughs have opened up new GPCR research fields exploring structural mechanisms and complexes, and novel principles to achieve a more selective action through ‘biased agonism’ preferentially activating a specific signal protein or ‘allosteric modulation’ of the natural ligand response from an alternative binding site. The GPCR database, GPCRdb (4–6) serves the wide GPCR community, currently ∼1800 monthly users, with reference data, web server analysis tools and dynamic visualisation of data and statistics. Current data ranges all 398 human non-olfactory GPCRs and 16 G proteins, over 14 000 species orthologues, 30 328 binding site mutations, all 218 experimental structures, and 10 059 extracted ligand interactions.

GPCR crystal structures have now been determined for the major human classes A–C, F (D and E fungal and amoeba receptors, respectively), but not yet for the Adhesion (B1) and Taste 2 classes, which have atypical pharmacology in that they are not activated by an endogenous agonist. In total, structures are available for 46 unique receptors, however close to 90% of the non-olfactory receptors still lack a crystal structure (http://gpcrdb.org/structure/statistics). Furthermore, only eleven receptors have a structure in the active state. Hence, many GPCR studies utilise homology models for virtual screening, lead optimisation, elucidation of ligand binding sites, off-target rationalisation, modeling of complexes, and molecular dynamics (7). The latest GPCR Dock assessment (8) showed that the accuracy of such models has greatly increased, and the best models utilize more than one template. Our 5-HT_1B_ model achieved the best RMSD compared to the crystal structure and was based on five backbone templates and a position-specific rotamer library derived from all GPCR structures. This strategy of *‘Less model—more Xtal’* covers more of the model with experimental data by extending the main template with alternative local templates for missing or distorted segments. Herein, we present the first resource with pre-generated multi-template models of all human non-olfactory GPCRs in their inactive, intermediate and active states (except for classes C and F for which active and intermediate templates are still missing).

GPCR ligands have previously been collated in a dedicated resource, GLIDA database (9), but maintenance was discontinued in October 2010 when the database contained 30,399 ligands. Today, the Guide to PHARMACOLOGY database offers curated reference ligands for a large number of protein families, including GPCRs (10), however this is just a small subset of the publically available ligands. The most comprehensive resource for biological activities is the ChEMBL database, which has compiled data from publications, patents and commercial sources (11). However, ChEMBL data is not presented and filtered to be directly accessible by many users. A further resource is the Ki database of the psychoactive drug screening programme containing broad target profiling, however most of which are single-point values (12). Herein, we present a new GPCR ligand resource that applies the same quality requirements as Guide to PHARMACOLOGY, but encompasses ligands from ChEMBL, GLIDA and the PDSP Ki database. It gives users novel possibilities, such as commercial availability, target selectivity and selection of ligands suitable for crystallography experiments.

## METHODS

### Technical features of the homology modeling pipeline

**Figure 1.**
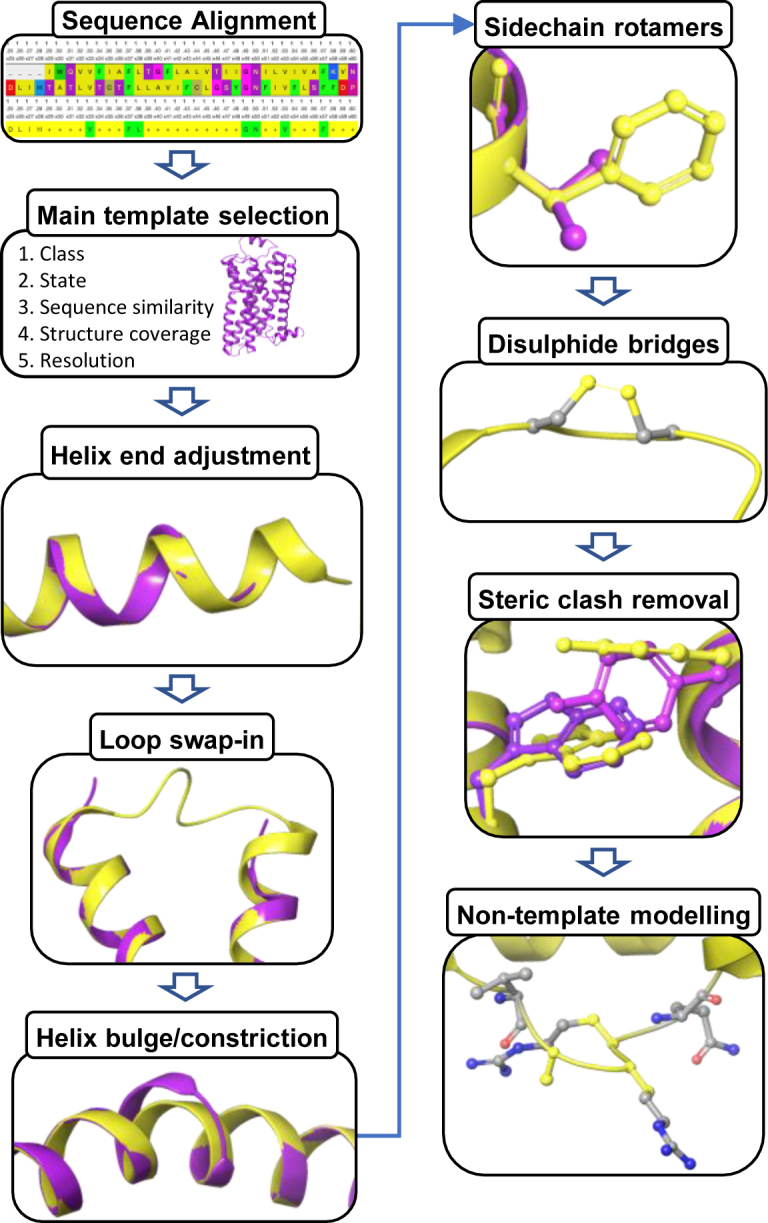
The GPCRdb homology model pipeline builds on the principle of *‘Less model – more Xtal’* meaning that more of the target is covered by structural templates, as an incomplete or partly distorted main template is complemented with alternative local templates. All templates and models are automatically updated upon each database update. Models are produced for all the human non-olfactory GPCRs in the inactive, and where templates exist (classes A and B1), also intermediate and active states.

The new GPCRdb homology modeling pipeline (Figure 1) updates the templates and models upon each database update. It was developed in Python in the Django web framework, utilizes several tools from Biopython (13), saves data in a PostgreSQL database, builds the final models with the help of MODELLER 9.18 (14) and uses NGL (15) for model visualisation. It leverages on integration of several unique manually annotated GPCRdb resources, including structure-based sequence alignments with generic residue numbers (16), GPCR structures (all in PDB) with defined preferred chain, helix segment borders, and distorted regions; and a position-specific sidechain rotamer library (17).

### Pairwise sequence alignment

Target-template pairwise sequence alignments are fetched from GPCRdb.

### Main template selection

For each receptor with a structure at least one (the most complete) representative template (currently totalling 169) has been manually annotated to define aligned segments (TM1–7, H8, ICL1–2 and ECL1–2), bulges, constrictions and any missing or distorted regions. A model main template is selected based on: (i) class (B2 to B1 and Taste2 to A), (ii) state (active/intermediate/inactive), (iii) receptor sequence similarity, (iv) structure coverage (no. generic residue positions (16) with coordinates) and (v) resolution. The template states are defined based on the extend of outward and inward movement of TM6 and TM5, respectively, on the intracellular, G protein binding side (http://docs.gpcrdb.org/structures.html#structure-state).

### Helix end adjustment

Transmembrane helix ends are in some places (typically in ICL3) altered due to protein fusions, H8 is sometimes missing or truncated, and both can be distorted due to crystal packing effects. Non-native parts of helices are removed from the initial model and missing segments are modeled based on a local alternative template, which is superposed onto the last helical turn of the main template.

### Loop modeling

As a unique feature, GPCRdb has alignments and generic residue numbers for loop regions with a conserved secondary structure; ECL1: three-residue turn motif with terminal amino acids packing between TM2-TM3, ICL1: single-turn helix, ECL2: cysteine forming a disulphide bridge to TM3 plus two following residues, and ICL2: two-turn helix. This allows our pipeline to model these loops based on alternative templates, where needed. Structurally disordered loops are also modeled based on an alternative template if the main template is incomplete, distorted or has a different length than the target. An alternative template is chosen based on length (as many residues as the target), sequence similarity, structure coverage and resolution. The template is superposed on the adjacent helix ends, except for ECL2 for which a first and latter half are anchored on the backbone of the conserved cysteine (position 45x50). If the main template structure lacks the backbone coordinates of the conserved ECL2 cysteine, these are inferred from an alternative template that is superposed using the last TM4 residue, first TM5 residue and the conserved cysteine in TM3 (3x25) that makes up the other half of the disulphide bridge. Altogether, this ensures that only a few remaining loop stretches need to be modeled without a template.

### Helix bulge and constriction treatment

When the target or template has a unique 7TM helix bulge or constriction (a local turn of π- or 3_10_-helix, respectively), a swap-in template with the same configuration as the target is applied. Such helix distortions are associated with characteristic sequence motifs, typically involving proline or glycine residues, and have been uniquely accounted in the GPCRdb alignments and residue numbers (16).

### Sidechain rotamer modeling

Amino acids not conserved between the target and main template are defined from a position-specific rotamer library extracted from all experimental GPCR structures (17). A conserved amino acid from the same structure/sequence position is superposed onto the backbone atoms of the original residue. In practice, this generates a chimeric structural template with a significantly higher sequence identity, often over 20%, to the target than a single template. Also, template mutations, introduced to facilitate expression and crystallization, are reverted to the wildtype residue using the rotamer library.

### Disulphide bridge introduction

Two conserved disulphide bridges are modeled: (i) one cross-class between the top of TM3 (3x25) and ECL2 (45x50) and (ii) another present in some class A receptors that links two cysteines in ECL3 or ECL3 to the top of TM6.

### Non-template-based modeling of remainder

Residues not covered by a structural template get their missing backbone and/or sidechains modeled using MODELLER (14), which also re-models non-conserved sidechains with a steric clash.

### Building of a GPCR ligand database with information about commercial availability

Ligands and biological activities were imported from ChEMBL_23 using the ChEMBL protein IDs and API (11). Records were filtered to only include the standardised/translated pChEMBL values and the binding and functional assay types, while excluding those with disqualifying statuses in ‘data_validity_comment’ and ‘activity_comment’ (see https://www.ebi.ac.uk/chembl/faq). Ligand chemical (molecular weight, no. hydrogen bond donors/acceptors and rotatable bonds) and commercial availability data (vendor and catalogue number) were retrieved from PubChem (18) after building a translation routine between ChEMBL and PubChem compound identifiers.

## RESULTS

### Recent resources for GPCR drugs, G protein coupling selectivity and natural genetic variants

Three recent high-impact publications have been accompanied with tailored resources that integrate biologically and clinically relevant data and let researchers make their own analyses online at GPCRdb. The first new section, ‘Drugs’, supplement an analysis of the new trends for GPCR drugs, targets and indications covering FDA-approved drugs and agents in clinical trials (1). The first item is Drug statistics concerning, e.g. targets, drug molecule types and modes of action, disease indications and clinical phase distributions. Secondly, a Drug target mapping page shows the distribution of in-trial and approved clinical agents onto a target tree classification of GPCR classes, ligand types and receptor families to give a rapid overview for targets of interest. Finally, a Drug browser allows for navigation and filtering of complete information for drugs, targets, indications, clinical progression and references.

The second new section, ‘Signal Proteins’, was added as part of a landmark study on GPCR-G protein selectivity (19). This features selection of human receptor sets by their GPCR-protein coupling data, G protein sequence alignments and residue topology diagrams, structures, interface mapping and search and in vitro mutations of GPCRs and G proteins with effect on signalling. The third new (sub)section Genetic variation (found in the Receptors section) was added as part of a comprehensive report on pharmacogenomics of GPCR drug targets (20). This subsection features Variation statistics with counts and densities of missense and loss of function polymorphisms across receptors, receptor families, ligand types and GPCR classes; a Receptor variant browser tabulating missense variant data and functional annotations (such as ligand and effector protein binding sites, post-translational modification sites, sodium ion pocket and micro-switches) by target(s) and mapping their topology in receptor residue diagrams; as well as an Estimated economic burden caused by genetic variation along with UK NHS sales and prescription information per drug.

### Ready-to-use models of several states for all non-olfactory human GPCRs

GPCRdb contains inactive state models of all human non-olfactory GPCRs and intermediate as well as active state models for classes (families) A (Rhodopsin), B1 (Secretin), B2 (Adhesion) and T (Taste 2). This includes also refined experimental structures filling in missing and replacing distorted regions and reverting mutated amino acids (added to the Structure Browser). Models are listed on the receptor pages, but also available from a new homology model browser (http://gpcrdb.org/structure/homology_models) allowing for multiple structures to be filtered, superposed, assigned generic residue numbers and downloaded. Finally, each receptor model has a page with embedded visualisation and details of which templates were utilised for which segment.

### Homology model accuracy

**Figure 2.**
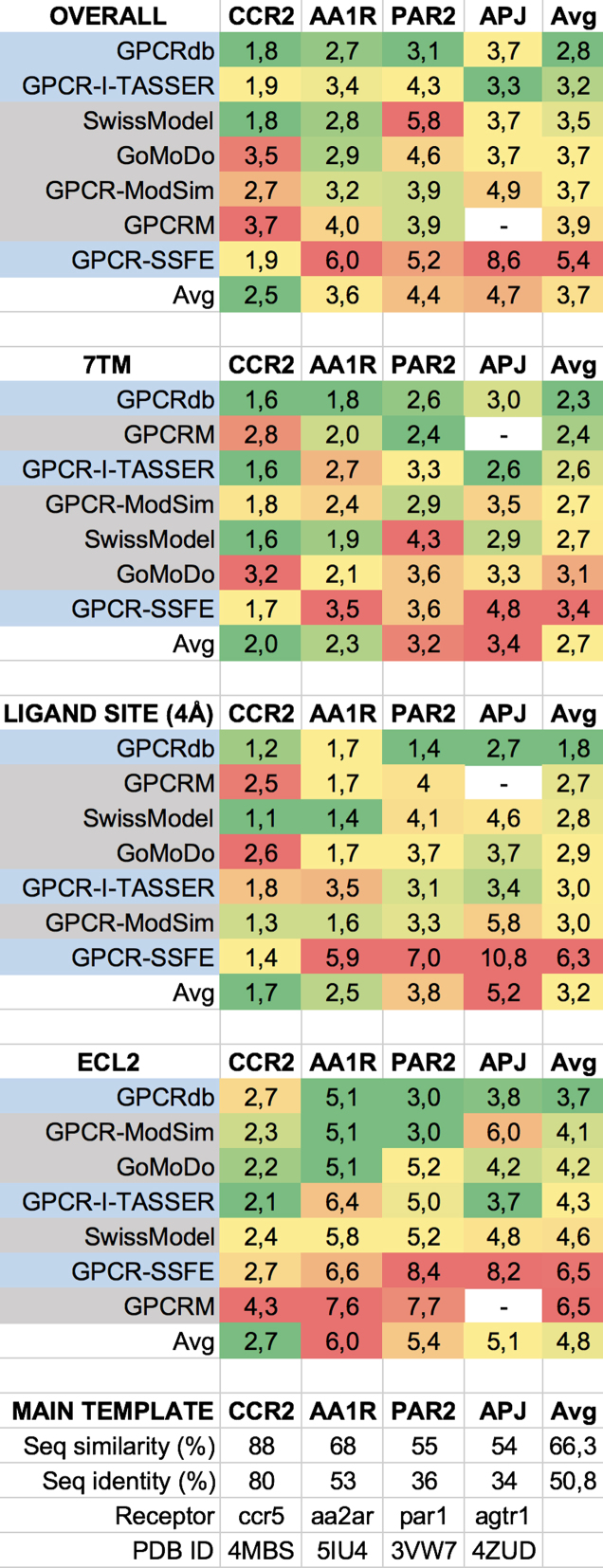
RMSD (Å) values of database/server homology models calculated directly upon release of the first structure for four GPCRs (AA1R: 5UEN, CCR2: 5T1A, PAR2: 5NDD and APJ: 5VBL). Notably, the GPCRdb homology models have the best average RMSD values in all four categories. Superimposition was performed on backbone heavy atoms. For comparability, RMSD calculations were restricted to residues present in all of the models. SWISS-MODEL settings: default, used main template listed on top. GOMoDo settings: Blast – 2 search rounds, MODELLER options—two models, global alignment (no realign templates), no loop refinement, chose model with best normalized DOPE score (PAR2 model was sixth best, ones before were incomparable due to a shift in sequence numbering). GPCRM settings (kindly provided by the developers): beta version (under development), ECL2 disulphide bridge specified, Task mode Auto, Set of templates inactive, Lysozyme do not add from template, Rosetta loop-modeling yes, fast, additional options left default. Selected r01 model. GPCR-ModSim settings (kindly provided by the developers): group inactive, alignment was edited, number of models 10, selected one with best DOPE score, no Lenard-Jones restraints, select top templates – default, ECL2 disulphide bridge specified, short loops ICL1, ECL1, ICL2 and ECL3 were modeled with loop modeling, number of models 5, selected one with best DOPE score, no MD. GPCR-i-TASSER model selection from repository: Model 1. GPCR-SSFE model selection from repository: EntireModel1, loop versions 0.

Figure 2 shows RMSD values of database/server homology models calculated directly upon release of the first structure for four GPCRs. The GPCRdb homology models have the best average RMSD values in all four categories: overall (2.8 Å), 7TM (2.3 Å), ligand site (1.8 Å) and ECL2 (3.7 Å). Among other databases with pre-generated models (blue background), GPCR-I-TASSER performs the best, especially in the overall and 7TM. In contrast, three modeling web servers (gray background) perform best in different categories; SWISS-MODEL: overall, GPCRM: 7TM and ligand site, and GPCR-ModSim: ECL2. As could be expected, the RMSDs of the overall and 7TM models correlate directly with the sequence similarities and identities to the main template (bottom of figure). Altogether, the comparisons confirm the technological advantages of the GPCRdb homology modeling pipeline, which gives all researchers access to ready high-quality models without the need for own configuration.

### Example model

**Figure 3.**
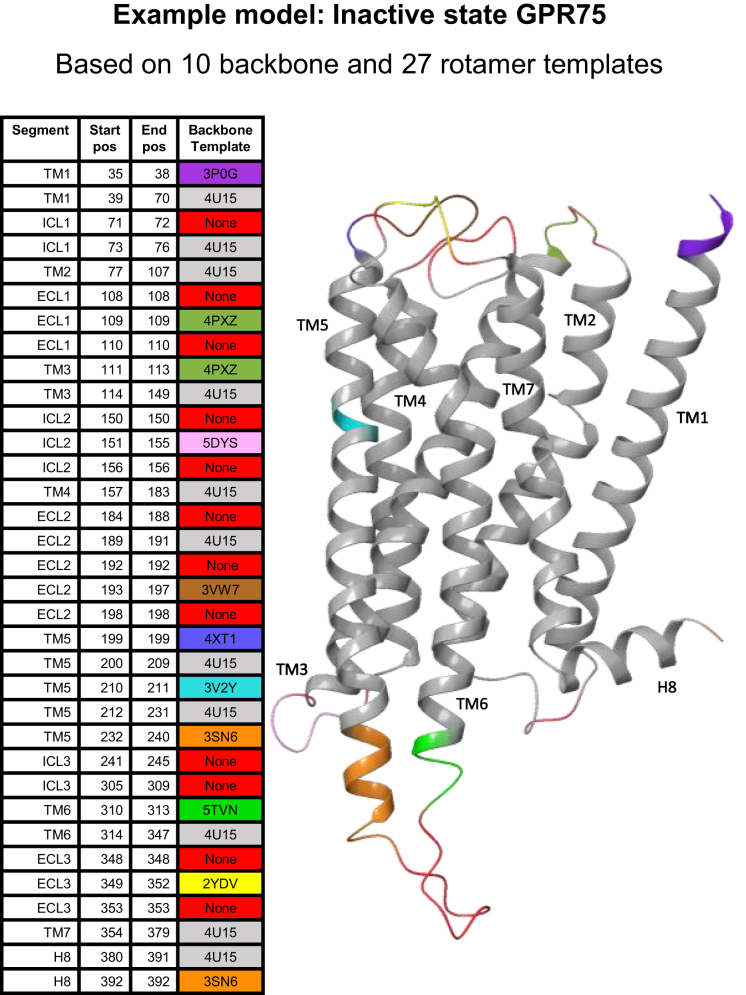
Example model of the inactive state GPR75. The colors indicate the use of 10 different backbone template to fill in lacking coordinates, elongate helices, add missing secondary structure segments to loops and to remove a main template helix bulge not shared by GPR75. Furthermore, the use of 27 sidechain templates from the GPCR position-specific rotamer library increased the percentage of residues that could be based on an identical amino acid from 15.7% to 58.2%.

Figure 3 shows an example model, the inactive state GPR75, which was based on 10 backbone and 27 sidechain templates. Alternative backbone templates were used to extend helices that were too short in the main template. These were TM1 start (3P0G), TM3 start (4PXZ), TM5 start (4XT1), TM5 end (3SN6), TM6 start (5TVN) and H8 end (3SN6). This feature is especially useful in the TM5-ICL3-TM6 region, where most GPCR crystal structures have non-native conformations due to fusion proteins. Alternative templates were also used for loops that differed in length or had missing coordinates. These were ECL1 (4PXZ), ICL2 (5DYS), ECL2 (3VW7) and ECL3 (2YDV). In TM5, an alternative template was used to remodel a main template bulge not present in the GPR75 alignment, as this does not share the associated sequence motif. Finally, by applying our GPCR position-specific rotamer library, the percentage of residues that could be modeled from an identical amino acid increased from 15.7% to 58.2%.

### The GPCRdb ligand database and statistics

**Figure 4.**
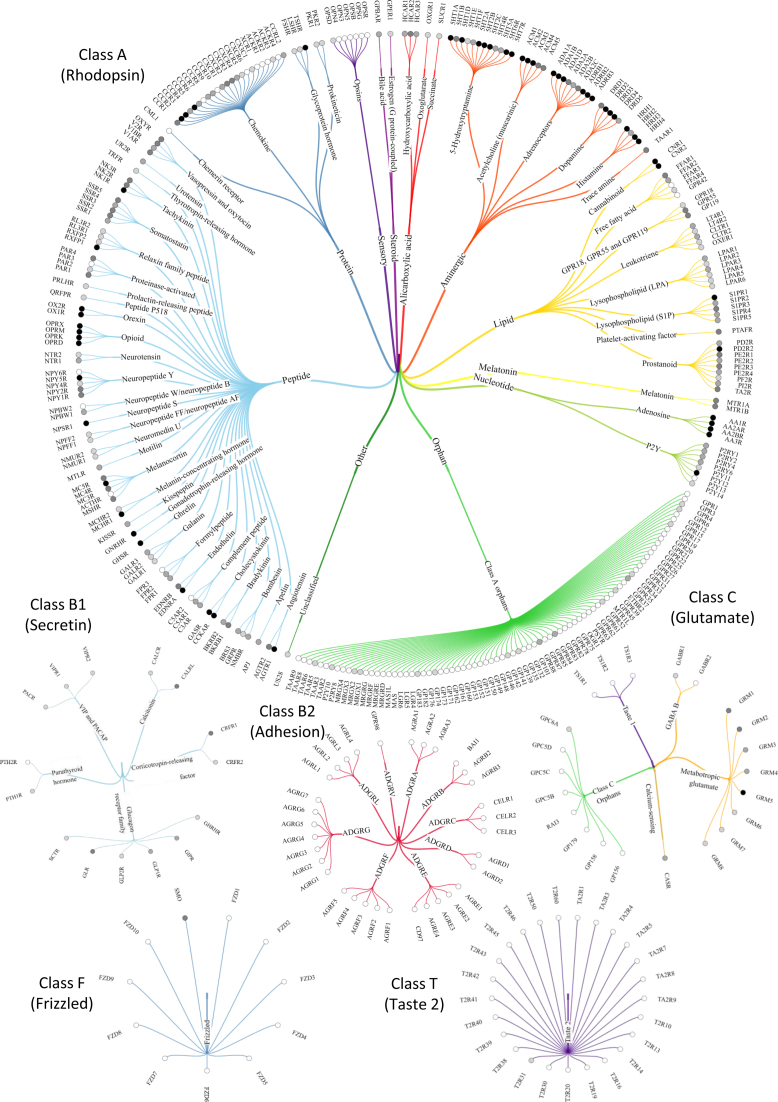
Trees showing the human GPCR classes (GRAFS family): A (Rhodopsin), B1 (Secretin), B2 (Adhesion), C (Glutamate), F (Frizzled) and T: Taste 2. Each tree is sorted alphabetically by ligand type (only class A) and receptor families which share the same physiological ligand. The tree branches are color-coded by ligand types, and the gray-scale circles before receptor names indicate their number of ligands (white: 0, light gray: >100, gray: >500 and black: >1000).

This first instalment of the GPCRdb ligand database contains 144 773 ligands, 597 targets, 29,530 assay experiments amounting to ∼320 000 activity records. A dedicated ligand statistics page has been added to provide continuously updated distributions of ligands across the GPCR classes. Overall statistics is provided in a table listing the numbers of: ligands, average ligands per receptor and receptors that have a ligand. Specific receptors and receptor families are shown in trees, as also illustrated in Figure 4. The vast majority of ligands (91%) are found for class A GPCRs with great abundance (>1000 ligands) for aminergic, adenosine, opioid receptors, but no or very few ligands for the many understudied orphan receptors.

### Ligand Browser

The ligand browser spans four separate pages for target selection, ligand filtering, ligand source data and commercial availability; all of which allow for filtering directly in the column headers. The first page allows for selection of targets on the level of receptors, receptor families or classes, while displaying the numbers of associated ligands. The second page shows the min, max and mean activities for ligand-receptor-species triads across all their experiments; hence provide a non-redundant overview suitable for filtering of ligands by their activities, commercial availability and chemical properties, such as logP. The negative logarithmic (p) values are used to compare the large span of affinities/activities, which are also provided in nanomolar units. A third page allows users to retrieve the exact ligand activities in all separate assay experiments. The fourth and final page adds vendor names and identifiers to facilitate ligand purchases. The three ligand-containing pages have the option to download ligands smiles (with ChEMBL identifies) and activity spreadsheets (csv format) to facilitate further processing.

## CONCLUSIONS AND OUTLOOK

In the last two years, GPCRdb has enabled and disseminated high-impact biological analyses (1,19,20), through resources that also allow scientists to analyse the data themselves. Herein, we have reported homology models of unprecedented quality for all human non-olfactory GPCRs and across activity states. The models will stay up to date, as the integrated pipeline incorporates new templates upon database re-building. The structural completeness and precise rotamers of the GPCRdb homology models make them uniquely suited for molecular dynamics and docking, respectively. The GPCRdb ligand database allows users to find relevant tool compounds and drugs faster and to know where to buy them, thereby facilitating experimental studies across many GPCR disciplines. Going forward it would be very valuable to expand from ChEMBL to additional ligand sources and to implement a ligand selectivity filter. Furthermore, the structural revolution within the GPCR field should now allow for analysis of the successful constructs and data-driven design toward more structures. Finally, GPCR signalling is a hot topic warranting additional resources, such as β-arrestin data, GPCR-G protein complex models and a database of published biased agonists.

## AVAILABILITY

GPCRdb is available at http://www.gpcrdb.org and can also be accessed via REST web services, as exemplified at http://docs.gpcrdb.org/web_services.html. The open source code and a virtual machine is available at https://github.com/protwis/protwis.
